# The impact of flipped teaching on EFL students’ academic resilience, self-directed learning, and learners’ autonomy

**DOI:** 10.3389/fpsyg.2022.981844

**Published:** 2022-12-05

**Authors:** Siros Izadpanah

**Affiliations:** Department of English Language Teaching, Zanjan Branch, Islamic Azad University, Zanjan, Iran

**Keywords:** academic resilience, autonomy, flipped learning, flipped teaching, selfdirected learning

## Abstract

**Introduction:**

This study attempted to investigate the impact of flipped teaching (FT) on EFL (English Foreign Language) students’ academic resilience (AR), self-directed learning (SDL), and learners’ autonomy (LA).

**Method:**

To do this, the researcher selected 354 participants by the two-stage cluster sampling method. This research was quasi-experimental based on the pretest, and post-test, with experimental and control groups. Three questionnaires were administered to collect data. The questionnaires were analyzed using SPSS 24 software and inferred analysis of covariance (ANCOVA).

**Results:**

The covariance study showed that FT significantly affected AR, SDL, and LA in learning with the help of the pre-test covariate variable (p<00.5). Also, the mean scores of students in the pre-test and post-test in the experimental group were significantly different. The mean scores of EFL students’ AR, SDL, and LA were higher through FT. It is suggested that school principals provide the ground for teachers’ participation in workshops on new teaching strategies so that teachers can benefit from new teaching approaches, including FT in the classroom.

**Discussion:**

The study results showed that the mean AR of students in the experimental group’s post-test compared to the pre-test in both groups has significantly increased. The research findings indicate a positive effect of the flipped class on the levels of SDL. Based on the results, the flipped lesson class approach significantly affected the LA of English language learners as a foreign language. The findings of this study confirm previous relevant studies on the impact of flipped course classes on the LA of English language learners as a foreign language.

## Introduction

Due to the progress of technology and science, the acceleration of the evolution and development of science and technology in the present age is very high and significant (Shakarami et al., 2017; Dogan et al., 2021). Researchers produce new technologies every day. Due to social and technological changes, the knowledge and science taught in schools and universities needs revision (Bolzani et al., 2021). In recent decades, with the dramatic changes and the expansion of science, the need to upgrade human knowledge, skills and develop lifelong learning skills is one of the primary goals of the educational system (Mirzaei and Hatami, 2019). With information technology and the increasing development of the educational system, it has shifted its activities to e-learning. The general popularity of this type of education is that some people ignore the benefits of traditional education and the interaction between teachers and students and consider e-learning as the only way of teaching for sustainable learning in formal education.

This increasing advancement in technology in the world has led to the fact that traditional teaching methods no longer meet the needs of students (Safari, 2020). Researchers have made many efforts to provide new and innovative approaches based on students’ needs to develop them as creators and producers of science and technology. In recent decades, we have also witnessed the progress of novel approaches to transferring delegated knowledge from a behavioral perspective to a constructivist perspective (Schultz et al., 2014; DeLozier and Rhodes, 2016; Bahmani et al., 2017).

Therefore, the researcher argued that flipped classroom education with all these benefits can significantly affect students’ academic resilience, self-directed learning, and learner autonomy because flipped education’s effectiveness in the classroom is unclear. Therefore, to respond appropriately to their primary concern, the researcher in this study tries to study the effectiveness of flipped education as an independent variable on the dependent variables of students’ academic resilience, self-directed learning, and learner autonomy. However, since the study results can explain the effectiveness of flipped education on students’ academic resilience, self-directed learning, and learner autonomy; the results can be a great guide for policymakers and educational designers.

An obstacle to learning a foreign language in Iran is communicating in the English language. However, English is part of the educational curriculum in guidance and high schools.

A problem that English learners often face is that modern technology is not sufficiently used in teaching English (Maniei, 2003; Lin et al., 2022). Two primary tools in teaching language can be the Internet and Educational videos; however, teachers do not use them enough in language classes (Fariborzi and Abu Bakar, 2011). So far, instructors have made various decisions and implemented new methods and approaches to improve the unfavorable conditions of foreign language teaching in Iran. Scholars have corrected many textbooks and educational programs by trial and error in teaching and learning English but achieving the desired goals in this field is not clear yet (Safari and Rashidi, 2015).

Over the past few decades, technological advances have introduced alternative forms of active learning with the challenge of effective foreign language teaching. These important technological advances include high-capacity Internet, cloud computing, video-sharing websites, and recent news publishing. The collaboration between effective active learning-teaching methods and technological advancements is the “flipped classroom” method. A type of instructional style involves transferring a lecture’s component to a lesson outside the classroom to incorporate other instructional activities during the classroom session (Strayer, 2012).

## Review of literature

### Flipped classroom

The flipped teaching method was introduced and developed in (2012) by two chemistry teachers, Bergman and Sam. Later, this approach gained credibility among researchers and experts. Like the traditional teaching method, the primary teaching philosophy of this method emphasized the principle of students’ homework.

The flipped classroom is an educational strategy and mixed learning. It turns education into a student-centered model in which the class examines topics more deeply and creates learning opportunities. In traditional education, lessons in the flipped classroom may include learning based on homework activities (DeLozier and Rhodes, 2016). On the one hand, students can spend more time in the classroom in this type of learning. They are more active in learning and creating knowledge. Meanwhile, they test and evaluate their knowledge (Thomas and Philpot, 2012).

Flipped teaching includes spending time in the classroom for individual learning and using different teaching and learning methods. Encourage learners to take responsibility for their learning (Helgeson, 2015). Content is set aside in the classroom, and teachers can provide classroom activities by teaching learners how to find the cause of problems and apply information in real life. The teacher and educational approach must be efficient and practical (Jahed Motlagh et al., 2015).

However, modern teaching approaches and methods keep track of learners’ needs to reinforce students’ engagement in the process of learning and create opportunities for mutual interactions. In addition, it is attempted to consider the learners’ interests, capabilities, differences, and affective factors to provide better facilitative instructional outcomes. This way of teaching creates an interactive and dynamic atmosphere in which learners actively engage in doing tasks (Du, 2021). Besides, it is required to keep up with the technological advancements that have been part of the language classroom to complete tasks and activities, support learners’ achievements, encourage learners’ engagement, and direct learners toward successful performance in the technology age (Sakulprasertsri, 2017). It has been said that Flipped Learning (FL) is a modern phenomenon in education that includes the changes in learner and teacher roles and the digital era.

### Academic resilience

Academic resilience means that students achieve good educational outcomes despite adverse conditions and challenges by changing existing behaviors or creating new behaviors, such as discipline, practice, or planning (Shakarami et al., 2017; Aliyev et al., 2021; Rich et al., 2022). Researchers argue that flipped teaching with all these benefits in the classroom can significantly affect students’ academic resilience because the effectiveness of flipped teaching in the school on students’ academic resilience is not apparent. There are no tools to measure the effectiveness of flipped teaching in the classroom.

Resilience is the ability to adapt to threatening situations, which means positive adaptation in response to unfavorable conditions. It significantly reduces students’ stress and increases students’ motivation to learn (Bahmani et al., 2017; Sahebyar et al., 2019).

Various studies show that students’ perceptions of teaching and learning activities are positive. They prefer visual classroom lectures but are more inclined to have more interactive classroom activities (Thomas and Philpot, 2012; Kavyani et al., 2015). Also, because the researcher in this study found that such research has not been done on female students in Zanjan, explaining the effectiveness of flipped teaching is essential to students’ academic resilience. In addition, the most crucial concern of researchers is that flipped teaching can transform traditional teaching methods (Bahmani et al., 2017) - develop critical thinking (Dehghanzadeh et al., 2018) – positively promote students’ creativity (Jafari et al., 2020), facilitate the learning of work (MobserMaleki and Kian, 2018) and technology lessons can be a practical comparison to conventional equipping methods and lead to learners’ academic achievement (Kavyani et al., 2015; Azimi and Bahmani, 2017). In the following, these articles discuss a few internal and external research that contribute to the quality of the study.

Nazaripour and Laei (2020), in a study investigating the effect of flipped learning (FL) on academic self-efficacy and learning mathematics of students with learning disabilities, found that FL is effective on academic self-efficacy and learning math lessons for students with learning disabilities.

Melissa (2020), in a study aimed at investigating “Facilitating student engagement through the FL approach in K-12: A systematic review,” found that the films produced by our respective teachers lead to more academic engagement in students.

Zamzami (2018), in a study, aimed to evaluate “Students’ learning performance and perceived motivation in gamified flipped-class instruction,” along with games based on the self-determining theory of receiving flipped education causes more motivation in students, and this leads to academic engagement and their participation will be in the classroom. Students, in particular, were so motivated that they competed with their classmates to the point where they were beaten. The findings led to the emergence of four important categories: 1-Motivation to learn before class. 2-Pre-class competition 3-Students ‘learning independence 4-Students’ participation or social interaction.

Ahanjan (2018), in a study aimed at investigating ‘academic achievement motivation and self-efficacy receiving flipped education in podcasting method based on model 5E (Engagement, Explore, Explain, Elaborate, and Evaluate) affect the academic achievement of students’ motivation and self-efficacy. The results showed that the podcast and the 5E model of academic achievement are effective, increase the motivation to study, and increase the involvement and self-efficacy of the subjects.

While learners have academic resilience, they can improve academic resilience skills by (a) associating new information with previous knowledge, (b) pondering on abstract and conceptual notions, (c) making use of particular strategies in completing tasks, and (d) perceiving their own opinions and thoughts (Hwang et al., 2019).

### Self-directed learning

The most important factor influencing students’ success and progress is the students themselves, whether the cause is internal or external (Jonathan and Aaron, 2016). In a world where conditions, technology, and science are changing rapidly, it’s important to have a comprehensive approach to content and how to learn it. Students must have appropriate learning skills, including self-directed learning (SDL) skills.

SDL is introduced as a process and learners with or without the help of others to identify learning needs, set goals, identify resources, select and implement necessary self-management plans (Field management including social environment of resources and facilities) with self-monitoring. The process by which learners become familiar with monitoring, evaluating, and formulating their cognitive learning strategies. SDL is a state of mind in which the learner feels individually responsible for their learning (Radnitzer, 2010; Khodaei et al., 2022).

SDL states that learners learn self-directed with their learning needs, setting goals, choosing a learning strategy, and evaluating the learning process results (Fisher et al., 2001; Hendry and Ginns, 2010; Bell, 2015). SDL increases learners’ confidence and capacity to learn independently in challenging educational and work environments. SDLis also an approach to the learning process that helps learners identify their own learning goals or needs through shared cognition and decision-making that makes a close and smooth partnership (Sarani and Aayati, 2014). Studies that have dealt with the effects of the flipped class include:

Kavyani et al. (2015), in applied research, have investigated the effect of flipped classes on the variables of academic self-regulatory academic achievement, group interaction, and students’ academic motivation. The statistical analysis results showed that the flipped class approach has a positive effect on all dependent variables.

Piri et al. (2018), in a study about flipped education, found that students who took the unit in flipped classes achieved better results in this course compared to the control group, and students in flipped classes performed better in solving problems, understanding educational concepts and content differed significantly from performing students who had traditional classes. Students’ feedback in flipped classes has also been positive, contributing to their efforts and conveying educational concepts.

Entezari and Javdan (2016), in a study about flipped class teaching in anatomy and physiology at Algardia College in New York claimed that the students’ performance in the exams and their satisfaction with the training course favored the students who had used the flipped class. They also concluded that flipped classes, combined with active learning strategies, were most effective.

### Learner autonomy

Some researchers have extensively used the Vygotsky theory-based framework for learning autonomy (Oxford, 2003; Abuhassna et al., 2022). A modification of Benson’s (1997) model refers to approaches based on Vygotsky’s theory of learning in which the social environment is highlighted. Asgari and Rahimi (2014) examined the effects of using a technology-based language learning framework on students’ perceptions or perceptions of the English classroom environment as a foreign language. They concluded that the language-based learning environment is more efficient, language-oriented, and facilitative than traditional teaching methods. Thus, using technology in the flipped class approach can increase language learners’ autonomy (Ankan and Bacall, 2011; Jarvis, 2013).

In addition, Hung (2015) concluded in his study that the FL approach improves students’ attitudes toward learning and their levels of participation. Zainuddin and Halili (2016) also conducted a content analysis of twenty reference articles on FL and concluded that most flipped class studies sought to examine students’ autonomous learning needs. In addition, Little (2022) hypothesized that the flipped class approach could address students’ need for autonomy, a sense of connection, skill, and efficiency. Davies et al. (2013) also pointed out that students in the flipped course can learn at their own pace, which increases their sense of autonomy. In addition, Rahman (2013) conducted a study on the relationship between CALL (computer-aided language learning) and autonomy of English language learners as a foreign language, which makes technology an important and efficient tool in learning, and concluded that computer-assisted language learning (CALL) has a positive effect on the autonomy of language learners if language learners find this helpful tool and take full advantage of it. In a similar study, Meri (2012) examined the relationship between learners ‘autonomy and CALL in Turkey, and her research showed that the CALL method promotes learners’ autonomous language learning. However, some studies, while demonstrating the benefits of learning environments using CALL to increase students’ autonomy and independence, also point to some of the limitations or problems associated with these environments. These findings confirm the concerns that the learner’s involvement in the computer-based approach does not necessarily lead to an increase in responsibility for learning management. For example, Kaur and Sidehu (2010) found that asynchronous online interactions *via* email could encourage autonomy in Malaysian university students. Still, more training in the optimal use of learning tools was needed to make the experience more efficient and effective. For this reason, in this study, we hypothesize that digital practice opportunities at home and outside the classroom can enhance learners’ autonomy and lead to greater individual responsibility for language learning.

Therefore, due to the volume of educational information and the short time for education, it was necessary to go beyond traditional methods and seek to create and strengthen academic skills in students, including skills: in academic resilience, self-directed learning, and learner autonomy in learning. Considering that there is little research in the database about the flipped classroom and, on the other hand, the importance of skills, academic resilience, self-directed learning, and learner autonomy in education and the need to teach English and the inefficiency and weakness of traditional methods in the process of teaching and learning, still more studies need to be conducted on these variables.

### This study investigates these hypotheses

1. Flipped teaching significantly affects the academic resilience of the eleventh of female high school students in language learning.
2. Flipped teaching significantly affects the SDL of the eleventh of female high school students in language learning.
3. Flipped teaching significantly affects the autonomy of the eleventh of female high school students in language learning.

## Materials and method

### Design of study and participants

This study was a quasi-experimental study with pretest, posttest, and control groups. It was conducted on 177 students in Zanjan, a city in northwest of Iran, in (2022). Inclusion criteria were female students who were studying in public high schools, in eleventh grade, and willing to participate in this study.

### Sample size

Considering the 95% confidence level (Z1-α = 1.96), the test power of 80% (Z1-β = 0.84) and based on the SDL variable in the studey of Soleymani et al. (2021) with the mean and standard deviation in the experimental group (M1 = 38.25 and S1 = 1.30), control group (M2 = 37.71 and S2 = 2.02), and using the formula for calculating the sample size in two independent groups, the total sample size was calculated to be 156 people. Taking into account the 10% drop in the sample, the final sample size was 177 participants in each group.

### Sampling method

The research setting was the public high schools of Zanjan, a city in the northwest of Iran. The research population included female students who were studying in the eleventh grade. In Iran, there are female and male schools separately. Zanjan city has 25 public high schools. Of these public high schools, 10 public high schools are for females’ students. The multi-stage sampling method was used for selecting participants. In the first stage, two public high schools were selected randomly. In the second stage, they were randomly divided into two experimental and control groups. In Zanjan, each public high school consists of 5 to 6 eleventh grade students that were studying in different fields. Also, each class has between 35 and 40 students. In the third stage, using the convenience sampling method 5 classes were selected for accessing to total sample size. A teacher who was fluent in flipped teaching was chosen to teach English.

### Procedure

In this regard, the educational content of the 11th-grade English course was prepared. After informing the experimental group about the purpose of the performance, the organized files were provided to them for 16 sessions. For this purpose, the teacher recorded 16 sessions of one and a half hours in audio and video for teaching in flipped teaching. With the help of an educational technologist, electronic content was prepared for each session. This electronic content was provided to the experimental group of students in a compact disc 1 week before the beginning of the classes and the relevant lesson plan. In addition, a virtual group consisting of teacher and students were formed in the Iranian social network. Students can access the teacher and ask them technical questions and problems during the program. By studying the lesson plan, students realized which electronic content and reference book pages they should read before each class to collect data from the standard questionnaire.

Data collection tools were standard academic resilience, self-directed learning, and autonomy questionnaires. These questionnaires were completed by the control and experimental groups in two pretest and post-test periods.

### Instruments

#### Academic resilience

Collect data from the standard questionnaire of academic resilience of Samuels (2004), which was standardized has three components: “communication skills,” “future and problem-oriented orientation,” and “positivity” and 55 items with a five-point Likert scale (from never with a score of 1 to always with a score of 5). Ten professors confirmed its face and content validity in the field of educational sciences. The reliability of the questionnaire was calculated through Cronbach’s alpha coefficient at a total of 0.81 (Omrani et al., 1400).

#### SDL questionnaire

A self-directed assessment questionnaire in student learning was designed by Fisher et al. (2001). This questionnaire consists of 40 questions that include three subscales. These subscales include self-control, willingness to learn, and self-management. This questionnaire has been standardized in Iran by Nadi and Sajjadyan (2011).

Shokar et al. (2002) obtained the validity of this scale by Cronbach’s alpha method for the whole test, 0.82, and for the self-management subscales, 0.78, 0.71 willingness to learn, and 0.60, self-control. In Iran, Soltani and Naeemi (2012) obtained the reliability of Cronbach’s alpha SDL questionnaire for the whole test, 0.92, and the self-management subscales, 0.85, 0.87 willingness to learn, and 0.84, self-control. Also, the construct validity and content validity of the questionnaire in this study were confirmed by three experts.

#### Autonomy questionnaire

Zhang and Li’s (2004) learner autonomy questionnaire consisted of 21 items. This questionnaire has two parts: the first part includes 11 items, and the second part contains 10 items. The first 11 items are in the form of a Likert scale and have five options ranging from “never” to “always.” The second part is in the form of multiple-choice questions, and participants must choose the closest answer to their beliefs and views or opinions between options. Participants are expected to answer questions within 33 min, with a maximum score of 105. Based on Zhang and Li’s (2004) design and using Cronbach’s alpha coefficient, the reliability of this questionnaire is estimated to be 0.80. In addition, Zhang and Li (2004) reported that this questionnaire also has high validity. The reliability of this tool in the present study has been estimated using Cronbach’s alpha coefficient of 0.61.

#### Cronbach’s alpha

Regarding the reliability of the questionnaires, Cronbach’s alpha index obtained for the questionnaires is expressed in Table 1.

**Table 1:** Cronbach’s alpha calculated for research questionnaires.

Questionnaires	Questions	Cronbach’s alpha
Autonomy	21	0.78
Resilience	40	0.84
Self-directed learning	55	0.79

The results showed that Cronbach’s alpha of autonomy, resilience, and SDL questionnaires were equal to 0.78, 0.84, and 0.79 and higher than 0.7, respectively, and the questionnaires had the necessary reliability.

### Statistical tests

This research used central indicators and dispersion such as mean and standard deviation to analyze the data in descriptive statistics. Univariate analysis of covariance was used in inferential statistics. Then it was analyzed using spss24 software and univariate analysis of covariance (ANCOVA).

## Results

### Inferential analysis

Multivariate analysis of covariance has been used to test the research hypotheses. Covariance analysis is a comprehensive form of analysis of variance. While comparing the means of one or more groups and estimating one or more independent variables, the effect of one or more intervening variables, or covariates, is excluded from the equation.

### Assumptions of analysis of covariance

Before analyzing the research data, the assumptions of the ANCOVA test, i.e., the normality of the data and the homogeneity of variance, are examined; the results of them are presented in the following tables:

#### Normality and homogeneity of variables

Data’s default normality was checked by the Kolmogorov–Smirnov test and variance homogeneity test with Leven’s test. The results are as follows:

According to the results of the Kolmogorov–Smirnov test in Table 2, the hypothesis of normality of research variables by control and experimental groups was confirmed (sig > 0.05). Also, according to Table 2, the Leven test accepted the hypothesis of homogeneity of variances (sig > 0.05).

**Table 2:** Data normality test and variance homogeneity.

		One-sample Kolmogorov–Smirnov Test				Test of homogeneity of variances			
		Experimental group		Control group					
Variables	Courses	Test statistic	*p* Values	Test statistic	*p* Values	Levene statistic	df1	df2	*p* Values
Autonomy	Pretest	0.13	0.200	0.09	0.200	0.39	1	258	0.532
	Post-test	0.11	0.200	0.09	0.200	0.02	1	258	0.868
Resilience	Pretest	0.16	0.087	0.11	0.200	1.81	1	258	0.184
	Post-test	0.09	0.200	0.11	0.200	0.09	1	258	0.758
Self-directed Learning	Pretest	0.13	0.200	0.09	0.200	3.59	1	258	0.067
	Post-test	0.10	0.200	0.13	0.200	3.59	1	258	0.066

### Assumptions 4 and 5: Regression slope homogeneity and confirmation of the effect of the auxiliary variable

The results were performed through an analysis of covariance, which is presented in Table 3.

**Table 3:** Results of analysis of covariance for academic resilience.

		Mean		Analysis covariance					
Variable		Experiment	Control	Type III sum of squares	df	Mean square	F	*p*-Value	Partial eta squared
Resilience	Pretest	158.80	161.03	36908.18	1	36908.18	699.446	0.000	0.73
	Post-test	183.03	160.46						

According to Table 4, the assumption of homogeneity of regression slope was accepted by analysis of covariance (*p* > 0.05). Based on the results of Table 4, the selection of the variable (pretest) as a covariate is confirmed in this study (*p* < 0.05).

**Table 4:** Reception of homogeneous regression slope.

	Reception of homogeneous regression slope			Correlation pretest and posttest		
	Variable	*F*	*p* Values	Variable	*F*	*p* Values
Hypothesis 1	Autonomy*group	1.32	0.23	Autonomy	526.50	0.000
Hypothesis 2	Resilience *group	0.57	0.52	Resilience	299.34	0.000
Hypothesis 3	SDL*group	0.72	0.33	Self-directed learning	260.84	0.000

### Investigation of research hypotheses

*Hypothesis 1*: Flipped teaching significantly affects the academic resilience of the eleventh of female high school students in language learning.

Analysis of covariance was used to test the above hypothesis. The necessary assumptions for the covariance study have been examined, and these assumptions are valid. The results of the study of covariance are recorded in the following tables:

As shown in Table 3, flipped teaching has a significant effect on resilience (*p* = 0.001, *F* = 699.44). Therefore, it was concluded that the mean of the two groups in the post-test after adjusting the pretest scores was significantly different from each other. As seen in the tables, the mean resilience scores in the control group in the pretest was 161.03, and in the post-test was 160.46, while the mean of this variable in the experimental group was 158.80 in the pretest and 183.03 in the post-test was reported. Due to the significant difference between the scores in the post-test in the control and experimental groups, it was concluded that by eliminating the pretest factor (Covariate), the flipped teaching increases resilience scores and according to the effect of the quadratic power factor of ETA 1 to 0.73, the resilience variability in the experimental group is due to flipped teaching.

*Hypothesis 2*: Flipped teaching significantly affects female high school students’ SDL of the language course.

Analysis of covariance was used to test the above hypothesis. The necessary assumptions for the covariance study have been examined, and these assumptions are valid. The results of the analysis of covariance are recorded in the following tables:

As shown in Table 5, flipped teaching significantly affects SDL (*p* = 0.001, *F* = 136.77). Therefore, it was concluded that the mean of the two groups in the post-test after adjusting the pretest scores was significantly different from each other. As can be seen in the tables, the mean scores of SDL in the control group in the pretest was 102.07 and in the post-test was 102.46, while the average of this variable in the experimental group in the pretest was 105.38 and in the post-test was equal to 113.38. Due to the significant difference between the scores in the post-test in the control and experimental groups, it was concluded that by eliminating the pretest factor (Covariate) of the flipped teaching approach, the learning scores of the SDL increase and according to the effect of the coefficient of quadratic power 34% of the variability of SDL in the experimental group is due to flipped teaching.

**Table 5:** Results of analysis of covariance for self-directed learning.

		Mean		Analysis covariance					
Variable	Course	Experiment	Control	Type III sum of squares	df	Mean square	F	*p*-Value	Partial eta squared
Self-Directed Learning	Pretest	105.38	102.07	4311.41	1	4311.41	136.77	0.000	0.34
	Post-test	113.38	102.46

*Hypothesis 3*: Flipped teaching has a significant effect on the autonomy of female high school students in learning the language course.

Analysis of covariance was used to test the above hypothesis. As observed, the necessary assumptions for the analysis of covariance have been examined, and these assumptions are valid. The results of the study of covariance are recorded in the following table.

As shown in Table 6, flipped teaching significantly affects autonomy (*p* = 0.001, *F* = 68.80). Therefore, it was concluded that the mean of the two groups in the post-test after adjusting the pretest scores was significantly different from each other. As seen in the tables, the mean scores of autonomy in the control group in the pretest was 58.88 and in the post-test was 59.84, while the mean of this variable in the experimental group in the pretest was 61.03 and in the post-test was equal to 65. 42. Due to the significant difference between the scores in the post-test in the control and experimental groups, it was concluded that the flipped education approach increases autonomy scores by removing the pretest factor (Covariate). Due to the magnitude of the effect of the ETA quadratic coefficient,1 to 21% of the autonomy variability in the experimental group is due to flipped teaching.

**Table 6:** Results of analysis of covariance for autonomy.

		Mean		Analysis covariance					
Variable		Experiment	Control	Type III sum of squares	df	Mean square	F	*p*-Value	Partial eta squared
autonomy	Pretest	61.03	58.88	939.54	1	939.54	68.80	0.000	0.21
	Post-test	65.42	59.84						

Tests of between-subjects effects.

## Discussion

### Regarding the first hypothesis

The study results showed that the mean academic resilience of students in the experimental group’s post-test compared to the pretest in both groups has significantly increased. Flipped teaching has improved students’ resilience because when educational materials are already available, they can listen and view the material repeatedly through audio and video. Once confronted with the teacher, they participate with great confidence with the teacher’s questions, and students are not only encouraged by the teacher but also get better grades in the same subject (Shakarami et al., 2017; Nazaripour and Laei, 2020; Aliyev et al., 2021). Flipped teaching greatly influences students’ future and problem-oriented orientation because future orientation is associated with positive outcomes that guide the person in the right direction to achieve predetermined goals and prevent deviation (Kavyani et al., 2015; Azimi and Bahmani, 2017; Rich et al., 2022). Problem-based learning, on the other hand, is a student-centered teaching technique in which students learn science by gaining experience and working together on a subject, while traditional teaching methods are school-based and in which learners are not allowed to think as a necessary thing in learning (Bahmani et al., 2017; Sahebyar et al., 2019; Melissa, 2020). If problem-based learning is accompanied by positivity, problem-solving will be achieved better because positive thinking removes fear and despair. With the trust and belief of his heart, he can achieve problem-solving.

Another study finding indicates a positive and significant relationship between the flipped education method and students’ academic resilience after implementing flipped teaching in the classroom. The academic resilience of 11th-grade female students in language lessons affects English. These findings are somewhat consistent with the results of Shakarami et al. (2017), Ahanjan (2018), Mirzaei and Hatami (2019) and Dogan et al. (2021). They also found that flipped teaching promotes a sense of school belonging and academic engagement. The flipped teaching method provides an active and interactive environment for students to learn, and the teacher acts as a guide and facilitator (Thomas and Philpot, 2012; Kavyani et al., 2015; Bahmani et al., 2017; Zamzami, 2018). As a result, students become actively and creatively involved in the subject matter because of engaging students. At the same time, teaching contributes to their academic achievement and helps manage the teacher’s classroom effectively.

### Regarding the second hypothesis

Since the variable of self-direction in learning is a general construct, the research findings indicate a positive effect of the flipped class on the levels of self-direction in education. The research results align with Kavyani et al. (2015) and Esmaeilifar et al. (2015).

Kavyani et al. (2015) showed that flipped teaching significantly affects academic achievement-academic self-regulation and students’ academic motivation. Ismailifar et al. (2021) also indicate that flipped classes strengthen students’ sense of belonging to school (Entezari and Javdan, 2016; Jonathan and Aaron, 2016). Radnitzer’s findings also indicate the positive effect of flipped classes on students ‘problem-solving ability. The findings of (Fisher et al., 2001; Hendry and Ginns, 2010; Bell, 2015) indicate that flipped classes significantly affect students’ attention and progress. In explaining the research findings, it can be said that the goal of all strategies and methods of teaching is students’ academic success. Flipped teaching has been considered an effective method in strengthening academic skills, including self-direction, due to the effective components in academic achievement and the emphasis on educational technology and individual skills in the rapidly changing world and information age. In addition to the effect of deep motivational learning on academic motivation, the flipped classroom can also be an atmosphere of cooperation with the previous preparation of students and create an optimal atmosphere in the classroom.

To promote literacy in middle school and high school, Khodaei et al. (2022) point out that one of them is self-motivated motivation and learning and its importance in learning and providing the education needed by the student for independent learning activities after graduation. This skill is especially effective in elusive courses such as English, which require further review and learning activities. This study revealed that the flipped classes could affect SDL skills, so it can be concluded that using the flipped classes, which emphasize the desire and individual differences - deep learning - the use of various educational software. Collaboration can provide the ground for students’ academic achievement by influencing, creating, and strengthening SDL skills, motivation, self-control, etc.

### Regarding the third hypothesis

This study sought to investigate the effects of the flipped course approach on the autonomy of English as a foreign language in Iran. Based on the results, the flipped lesson class approach significantly affected the autonomy of English language learners as a foreign language. The findings of this study confirm previous relevant studies on the impact of flipped course classes on the autonomy of English language learners as a foreign language. Zainuddin and Halili (2016) analyzed 20 reference articles on FL and concluded that most studies on following and assess the students’ autonomous (independent) learning needs. Abeysekera and Dawson (2015) also hypothesized that the flipped course approach could meet students’ need for autonomy, a sense of connection, skill, and efficiency. Davies et al. (2013) and McGivney-Burelle and Xue (2013) also point out that students in flipped classes can learn at their own pace, enhancing their sense of autonomy. A research study in Iran showed that technology affects the autonomy of language learners (Oxford, 2003; Ebrahimi et al., 2013). In addition, the results of this study confirmed the effectiveness of active learning and the active participation of language learners in the learning process, as they were not merely passive recipients of knowledge but took responsibility for their learning. In addition, they were conducting flipped course classes before the course allowed students to research and learn the subject at their own pace. McDonald and Smith (2013) stated that students are more active in implementing the flipped lesson method, facilitating an effective learning process. In addition, providing pre-class content and activities to students made them responsible for learning and reduced wasted time in traditional education (Baepler et al., 2014; Basal, 2015; Abuhassna et al., 2022).

These findings can also be attributed to the fact that students in the flipped teaching model have more freedom and flexibility to choose their preparation methods for class (Ankan and Bacall, 2011; Fulton, 2012; Jarvis, 2013; Little, 2022).

In this way, students can feel more confident and participate in the class, improving their English communication skills by performing various communication exercises and assignments. In addition, flipped course implementation provides students with a time-and place-independent study environment such as distance learning systems. Similarly, Hamdan et al. (2013) emphasized that implementing the flipped lesson course provides a flexible study environment for language learners. Extracurricular learning is flexible and can take place at any time and place according to the choice of language learners and following their level of education and individual needs (Davies et al., 2013). On the other hand, the authors believe that the flipped lesson class does not promote learning, and the results can be even worse than teaching and learning in a traditional educational context. Springen (2013) is one of the authors who has criticized this pattern and style of teaching, believing that the flipped course is over-emphasized and is just a “fleeting fashion” that does not increase students’ grades and learning. Atteberry (2013) questioned the effectiveness of the flipped class for second language learners (L2) and argued that this approach should be devoted to teaching and learning procedural knowledge. They stressed that students might be stubborn and come to class unprepared. Lecture videos should also be carefully prepared to prepare students for the course. Making such high-quality videos is difficult and time-consuming. Springen (2013) stated that the training plan templates in this approach are limited. Also, the flipped lesson is the biggest problem for teachers not preparing and publishing lecture videos but organizing in-class activities and including them in the class approach. Contrary to popular belief, this method does not lead to the training of responsible and independent learners but rather reduces their responsibility and increases the duty and responsibility of teachers (LaFee, 2013).

## Conclusion

This study aimed to study the effect of using flipped classrooms on EFL students’ academic resilience, self-directed learning, and learner autonomy of the eleventh female students in Zanjan city in an English language course. Findings from the analysis of covariance showed that flipped teaching could have a significant effect on the variables of academic resilience, self-directed learning, and learner autonomy – learning with the help of the pretest variable. Also, the mean scores of students in the post-test of the experimental and control groups were significantly different. The mean scores of academic resilience, self-directed learning, and learner autonomy were higher for those trained through flipped education.

Teachers’ teaching approaches play an important role in encouraging learners to adopt the best learning method. On the other hand, flipped teaching provides a suitable environment for students to relax without stress and anxiety and confidently enter the classroom and participate in class activities.

Therefore, considering the effectiveness of flipped education on students’ academic resilience, self-directed learning, and learner autonomy, it is recommended that teachers use this method of education in their teaching. School principals should make the necessary books on flipped education and these variables academic resilience, self-directed learning, and learner autonomy and strengthen them in libraries and available to teachers. They should hold specialized workshops to provide teachers with the benefits and introduce the flipped method. School principals should encourage teachers who use flipped classroom teaching. Teachers should also create a positive psychological atmosphere, and students can attend the classroom more calmly. Further investigation and experimentation into flipped teaching are strongly recommended.

### Some limitations of the study are stated here

First, the number of research questions was limited to three due to a lack of time. Second, the target population only included EFL learners from one city in Iran. Third, the sample size might threaten the generalizability of results. Another limitation of the study was the instrument used. In the present study, questionnaires were administered to collect data. Further studies could apply various data collection methods, such as observation, interview, diaries, etc., to triangulate data and gain more reliable and valid results.

### Suggestions for future studies

This study applied the flipped-classroom approach to investigate the impact of flipped teaching on EFL students’ academic resilience, self-directed learning, and learners’ autonomy. Future studies might work on the issue using different methods. Questionnaires were the only means to investigate the impact of FL on EFL learners’ students’ academic resilience, self-directed learning, and learners’ autonomy. In forthcoming studies, researchers could apply methods like observation, journals, interviews, and triangulation to provide more generalizable data.

In the present study, the impact of FL was investigated on each variable separately. The relationship between FL and other variables, students’ academic resilience, self-directed learning, and learners’ autonomy could be scrutinized through models.

Also, future studies might examine the problem in terms of other demographic variables, such as gender, age, and language level. This study was conducted in language institutes. It should be replicated in different contexts, such as private schools and universities, where students might present different perceptions of the questionnaires based on their conditions and needs.

## Data availability statement

The raw data supporting the conclusions of this article will be made available by the authors, without undue reservation.

## Ethics statement

Ethical review and approval was not required for the study on human participants in accordance with the local legislation and institutional requirements. Written informed consent from the patients/ participants or patients/participants legal guardian/next of kin was not required to participate in this study in accordance with the national legislation and the institutional requirements.

## Author contributions

The author confirms being the sole contributor of this work and has approved it for publication.

## Conflict of interest

The author declares that the research was conducted in the absence of any commercial or financial relationships that could be construed as a potential conflict of interest.

## Publisher’s note

All claims expressed in this article are solely those of the authors and do not necessarily represent those of their affiliated organizations, or those of the publisher, the editors and the reviewers. Any product that may be evaluated in this article, or claim that may be made by its manufacturer, is not guaranteed or endorsed by the publisher.
